# Synthesis of zirconium modified FDU-12 by different methods and its application in dibenzothiophene hydrodesulfurization†

**DOI:** 10.1039/c8ra05032e

**Published:** 2018-08-02

**Authors:** Qian Meng, Peng Du, Bo Wang, Aijun Duan, Chunming Xu, Zhen Zhao, Cong Liu, Di Hu, Yuyang Li, Chengkun Xiao

**Affiliations:** State Key Laboratory of Heavy Oil Processing, China University of Petroleum Beijing 102249 P. R. China duanaijun@cup.edu.cn xcm@cup.edu.cn

## Abstract

Zirconium modified mesoporous materials were successfully synthesized by different methods including direct synthesis and post synthesis (grafting and impregnating). Meanwhile, the corresponding catalysts were prepared. A series of techniques, including small-angle X-ray scattering (SAXS), transmission electron microscopy (TEM) and pyridine adsorption Fourier-transform infrared (Py-IR), were used to characterize the properties of supports and catalysts. The results of SASX and TEM characterization proved that all the modified materials retained the well-ordered mesoporous structure from the FDU-12 material. N_2_ adsorption–desorption results showed that the Zr-FDU-12 material obtained by direct synthesis (Zr-in-F) possessed higher specific surface area (709 m^2^ g^−1^), pore volume (0.65 cm^3^ g^−1^) and larger pore size (18.7 nm) than the Zr-FDU-12 materials obtained by the post synthesis. According to the characterization result of UV-Vis, Zr-in-F exhibited better dispersion of Zr species than materials obtained by the post synthesis. It was found that the incorporation of Zr species not only increased acidities but also enhanced the sulfidities of the Mo species. All the catalysts were evaluated for their activities in the hydrodesulfurization of dibenzothiophene, and the NiMo/Zr-in-F catalyst exhibited the best catalytic performance (97.3%), attributed to its higher specific surface area, larger pore size, higher sulfidity, and more acidic sites.

## Abstract 2

Zirconium modified mesoporous materials were successfully synthesized by different methods including direct synthesis and post synthesis (grafting and impregnating).

## Graphical abstract

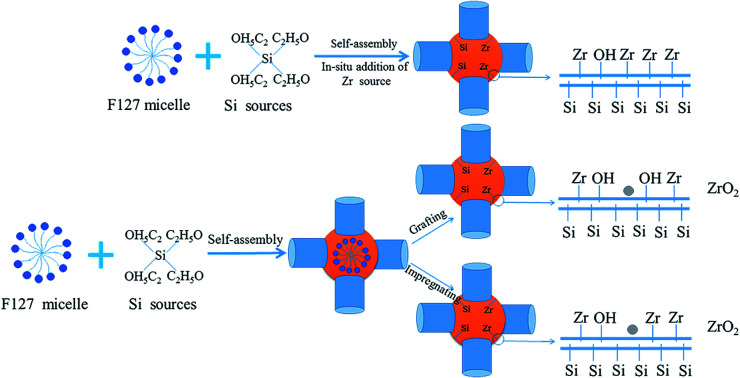

## 1. Introduction

With the legislation of environmental protection becoming more severe, vehicle fuel specifications have been published in many developed countries, especially for the control of sulfur content to near zero.^1–4^ As far as we all know, sulfur-containing compounds in oil can be removed by many techniques, including hydrotreating, adsorption desulfurization, oxidative desulfurization and biological desulfurization processes, in particular the hydrotreating process is currently the most used in the refinery industry.^5^ Catalysts usually play a significant role in the hydrotreating process, and their excellent physico-chemical properties can help active metal dispersion and promote the diffusion of the reactants and products. As the typical refractory molecule, 4,6-DMDBT has apparent steric hindrance, due to the adjacent substitutes of methyl at 4 and 6 positions, it is difficult for it to be removed over the traditional catalysts under the typical operation conditions. Thus the development of catalysts with high activity will be an effective thrust toward hydrodesulfurization.^6,7^

The activity of catalysts is restricted by many factors, among which the support contributes greatly, thus the development of new catalysts greatly depends on the supports. Al_2_O_3_,^8,9^ the representative of the traditional supports, possesses many good properties, such as excellent mechanical strength and low-price, however, its strong metal and support interaction (MSI) influences the aggregation states of active metals, then hinders the formation of type II-active phases with suitable stacks.^10^ Nowadays many carriers, including ZrO_2_,^11,12^ TiO_2_,^13,14^ and SiO_2_ (ref. 15–17) are used in hydrodesulfurization. Interestingly, mesoporous silica materials with high specific surface area, large pore volume and uniformly adjustable aperture (2–50 nm), attract much more attention of the scientific researchers, of which the advantages apparently make them to be the superior candidates in the field of adsorption, catalysis and biomedicine. With the application of mesoporous silica materials, however, the apparent limitation of the mesoporous materials reveals their low hydroxyl density on the surface, which makes a weak interaction between support and active metal, leading to inhomogeneous dispersion of active species. In order to improve the surface property of mesoporous silica materials, the researchers tried to embed the heteroatoms into the framework of mesoporous silica materials, such as Al^3+^, Zr^4+^ and Ti^4+^.^18–21^

It was found that the incorporation of heteroatoms was executed by two main methods: direct synthesis and post synthesis (grafting or impregnating).^21–26^ The direct synthesis shows the advantages of: (1) better dispersion of heteroatoms; (2) the better connectivity of the pores. Li *et al.*^22^ reported a direct synthesis of mesoporous material Zr-SBA-15 using cationic hexadecyltrimethylammonium bromide and non-ionic triblock copolymer Pluronic (P123) as the templates. According to the characterization results, Zr species were fully dispersed in the structure of SBA-15 when Zr/Si molar ratio is lower than 0.68. Olivas *et al.*^23^ recently obtained Ti-containing and Al-containing SBA-15 by a direct-synthesis method, and the result showed most Ti species and Al species were incorporated into the framework of mesoporous SBA-15. The modified SBA-15 material had little difference with the pure SBA-15 material in specific surface area, volume and pore size, which indicated a good retaining of the connectivity of mesopore channels. However, there are two mainly challenges in the process of direct synthesis: (1) the local distortion of framework due to the effect of Zr species on the assembly of silicon species. (2) The difficult formation of the Me–O–Si bond. Since the post synthesis method is to process the materials that have been prepared, thus it can avoid the problem of framework distortion.^24^ Nevertheless, the apparent disadvantages also need to be noted: some isolated external-skeleton species form to the small particles in the post process, which result in the blockage of the pores channels, consequently decreasing the specific surface area and volume. Klimova Tatiana *et al.*^25^ successfully synthesized a series of SBA-15 modified with aluminum oxide (Al_2_O_3_) or transition metal oxides (TiO_2_, ZrO_2_) by chemical grafting. It was found that the surface area and pore volume of the material were greatly reduced after modification, especially for the transition metal oxides. Wang *et al.*^21^ recorded the same phenomenon in the process of synthesizing Zr-MCFs material, especially at a low ratio of Si/Zr, which was ascribed to the formation of ZrO_2_ particles.

The excellent HDS catalysts should have not only the well dispersion of the active metals, but also the excellent mass transfer performance. Zhao Dongyuan group firstly synthesized three-dimensional highly ordered face-centred cubic (*Fm*3*m*) mesoporous silica material FDU-12 using triblock copolymers (F127) as the template,^27^ of which the structure was very favorable to the diffusion of reactants and products. In previous research, Cao *et al.*^28^ reported the synthesis of Ti-FDU-12 through a two-step method of prehydrolysis of TEOS followed by addition of titanium butoxide as a titanium precursor, and the catalyst with the Si/Ti ratio of 5.0 exhibited the highest catalytic performance in hydrodesulfurization of DBT and 4,6-DMDBT. Wang *et al.*^29^ synthesized different morphologies FDU-12 materials using different inorganic salts as the additives, and they all exhibited better catalytic performance in hydrodesulfurization with DBT as the probe molecules. It is clearly identified that FDU-12 has great potential to be the catalyst support for hydrodesulfurization.

In the present work, a series of mesoporous Zr–F materials were successfully synthesized with a Si/Zr molar ratio of 20 through three different methods, including the direct synthesis, grafting and impregnation. The corresponding NiMo/Zr–F catalysts obtained by different modified methods were prepared by a two-step incipient wetness method. The results of variable characterization methods demonstrated that all the as-synthesized materials kept better ordered mesoporous structures. The catalytic performances of the catalysts were evaluated on the hydrodesulfurization using DBT as probe molecule under 613 K, 4 MPa and different WHSVs (20–150 h^−1^). The contributions of different methods to incorporate Zr into the catalysts were discussed in detail.

## 2. Experimental

### 2.1 Synthesis of materials

Mesoporous FDU-12 material was synthesized using nonionic triblock copolymer (F127, Sigma Aldrich) as structure-directing agent, 1,3,5-trimethylbenzene (TMB, Aladdin) as the pore expanding agent, and tetraethylorthosilicate (TEOS) as the silica source. Furthermore, the incorporation of Zr to the support materials was realized by three different methods, involving direct synthesis, grafting and impregnation methods, of which zirconium oxychloride octahydrate (ZrOCl_2_·8H_2_O) was taken as Zr source. All the chemical agents were used directly without further purifications.

Pure mesoporous silica FDU-12 was prepared according to the method reported in the literature.^27^ Typically, 2.0 g of F127 and 5.0 g of KCl were dissolved in 120 ml of 2.0 M HCl with stirring until the mixture became a clear solution. Then, 2.0 g of TMB was added into the above solution. The mixture was stirred at 288 K for 24 h before 8.3 g of TEOS was added. Afterwards, the mixture continued to be stirred for 24 h, and heated statically at 373 K in a Teflon autoclave. The solid product was obtained by filtering with distilled water, drying at 373 K in air, and calcined at 823 K for 6 h to remove the templates.

#### Direct synthesis

Zirconia-modified FDU-12 with a specific Si/Zr molar ratio of 20, noted as Zr–F, was hydrothermally synthesized through the direct methods.^22,23^ The detailed procedure of the synthesis is as follows: firstly, added 2.0 g of F127, 5.0 g of KCl and 2.0 g of TMB into 120 ml of 2.0 M HCl, then stirred for 24 h to form a homogeneous solution; secondly, added 8.3 g of TEOS into the solution and stirred for 2–4 h. Afterwards, 0.64 g of Zr source was added into the mixture and stirred vigorously for further 24 h. Finally, the mixture was transferred into a Teflon autoclave and heated at 373 K for another 48 h. The product was obtained by filtering, washing with distilled water, drying at 373 K in air, and calcined at 823 K for 6 h. The as-prepared sample was denoted as Zr-in-F.

#### Post synthesis

The Zr–F materials prepared by the post synthesis methods, including (grafting and impregnation) were described as below.^21,24,25^ The mass ratio of the Zr source to the support is kept at 0.270.

##### a Grafting

At first, Zr source was dissolved in absolute ethanol and stirred at room temperature. Then, the pure FDU-12 support was added into the above solution with stirring at 353 K for 4 h. Afterwards, the mixture was filtered, washed with distilled water, dried at 353 K for 12 h, and calcined at 823 K for 6 h. Then, the obtained sample was denoted as Zr-gra-F.

##### b Impregnating

Firstly, the pure FDU-12 material was dried at 373 K for 3–4 h in order to remove the water adsorbed in the pores. Then, Zr source ethanol solution, of which the volume was equal to the pore volume of the material, was added dropwise into the dried support by the incipient wetness method. After ultrasonic treatment for 15 min, the solid was dried at 353 K for 12 h, and calcined at 823 K for 6 h. Finally, the product obtained was denoted as Zr-im-F.

### 2.2 Catalysts preparation

The supported catalysts NiMo/Zr–F (including NiMo/Zr-in-F, NiMo/Zr-im-F and NiMo/Zr-gra-F) with different modified methods were prepared *via* a two-step incipient wetness method. The heptamolybdate tetrahydrate ((NH_4_)_6_Mo_7_O_24_·4H_2_O) and nickel nitrate hexahydrate (Ni(NO_3_)_2_·6H_2_O) were used to be Mo and Ni precursors respectively, with the loadings of 12% MoO_3_ and 3% NiO in series impregnation. After each impregnation step, the sample was treated using ultrasonic for 15 min, dried at 373 K for 6 h, and calcined at 823 K for 6 h. Afterwards, the catalysts were obtained, donated as NiMo/Zr-in-F, NiMo/Zr-gra-F and NiMo/Zr-im-F respectively. In order to compare with the modified catalysts, NiMo/FDU-12 and NiMo/Al_2_O_3_ catalysts were also prepared to be the reference catalysts.

### 2.3 Characterizations of supports and catalysts

Small-angle X-ray scattering (SAXS) patterns were tested on a Nano STAR Small-Angle X-ray scattering system (broker, Germany) using Cu Kα radiation (40 kV, 35 mA).

Fourier transform infrared spectroscopy (FTIR) absorbance spectra were performed with a FTS-3000 spectrophotometer, and the wave numbers ranged from 4000 to 400 cm^−1^. The solid samples were detected after mixing with dry KBr.

Nitrogen sorption isotherms of the supports and catalysts were detected by a Micromeritics TriStar II 2020 porosimetry analyzer at 77 K. Using the Brunauer–Emmett–Teller (BET) method, the specific surface areas of the samples were calculated. The total volumes of mesopores and micropores were deduced from the amounts of nitrogen adsorbed at *P*/*P*_o_ = 0.98. According to the desorption branches of the isotherms using the Barrett–Joyner–Halenda (BJH) method, the distribution of pore size was obtained.

Scanning electron microscopy (SEM) images of the samples were recorded with a Cambridge S-360 apparatus operating at 20 kV. Transmission electron microscopy (TEM) images were detected using a JEOL JEM 2100 electron microscope operated at an accelerating voltage of 200 kV. The samples were grinded in an agate mortar and suspended in ethanol with ultrasonic. Several drops of the supernatant liquid were dripped on a copper grid coated with a sputtered carbon polymer.

The amounts and types of the acids on the surface of the samples were analyzed by a pyridine-FTIR (Py-FTIR) spectroscopy on a MAGNAIR 560 FTIR instrument with a resolution of 1 cm^−1^. The samples were dehydrated at 873 K for 5 h under a vacuum of 1.33 of10^−3^ Pa, and then adsorbed of the purified pyridine vapor at room temperature for 20 min. The IR spectra were recorded after the system was degassed and evacuated at different temperatures.

With a UV-Vis spectrophotometer (Hitachi U-4100) equipped with the integration sphere diffuse reflectance attachment, the UV-Vis diffuse reflectance spectroscopy (UV-Vis DRS) was detected in the wavelength range of 200–800 nm.

Raman spectra were recorded using a Renishaw Micro-Raman System 2000 spectrometer with spectral resolution of 2 cm^−1^. The 325 nm line from a He/Cd laser was to be the exciting source with an output of 200 mW. The Raman spectra between 200 cm^−1^ and 1200 cm^−1^ were automatically recorded at room temperature with the condition of 50 s accumulation at a 1 cm^−1^ resolution.

The X-ray photoelectron spectra (XPS) of the sulfided catalysts were tested in a Thermo Fisher K-Alpha spectrometer using Al Kα. All the obtained data were calibrated by taking the C 1s spectrum (binding energy = 284.6 eV) as a standard.

### 2.4 Catalytic activity measurement

The catalytic performances of all the catalysts (0.5 g, 40–60 mesh) were studied with using DBT as the probe molecule in a continuous flowing tubular fixed-bed reactor (8 mm inner diameter and 400 mm in length). In order to improve catalytic activity, all the catalysts needed to be presulfided for 4 h with H_2_ and 2 wt% CS_2_ dissolved in cyclohexane at 613 K and 4 MPa. After the above operation, the HDS reaction of DBT was carried out under the conditions of 613 K, 4 MPa, 200 ml ml^−1^ (H_2_/hydrocarbon) and different WHSV of 20–150 h^−1^. The sulfur contents of the feed and products were gauged with a sulfur and nitrogen analyzer (RPP-2000SN, Taizhou Central Analytical Instruments Co. Ltd., P. R. China). The catalytic performances (HDS efficiency (%)) of the catalysts were calculated by eqn (1):

1 HDS (%) = (S_f_ − S_p_)/S_f_ × 100%

where the S_f_ stands for the sulfur content of the feed, and S_p_ represents the sulfur content of the products.

## 3. Results

### 3.1 SAXS characterization of the supports

**Fig. 1:**
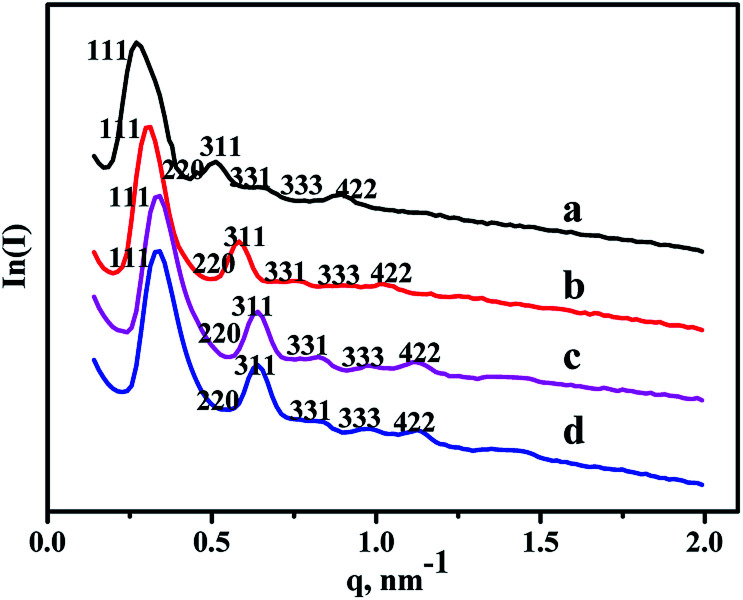
SAXS patterns of the as-synthesized materials: (a) FDU-12, (b) Zr-in-F, (c) Zr-gra-F, (d) Zr-im-F.

SAXS characterization results of all the materials are shown in Fig. 1. As can be seen from the patterns, six diffraction peaks existing in the pure FDU-12, belonged to different lattice planes, including (111), (220), (311), (331), (333), and (442).^30^ And Zr–F materials obtained by three different synthesis methods display the same diffraction peaks, illustrating that all the modified materials retain cubic *Fm*3*m* structure very well even after the incorporation of Zr. Furthermore, the intensities of all the diffraction peaks are relatively obvious indicating that all as-synthesized materials have better crystallinities after the modification of Zr. In addition, the peaks positions shift toward high angle, indicating the decrease of pore sizes after the incorporation of Zr species, and this was confirmed by the following N_2_ adsorption characterization.

### 3.2 N_2_ adsorption–desorption characterization of the supports

**Fig. 2:**
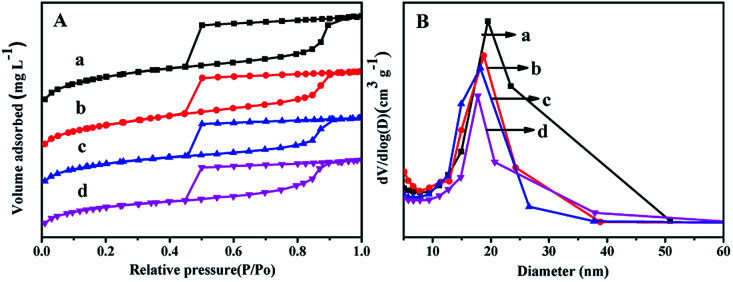
N_2_ adsorption–desorption isotherms (A) and pore size distributions (B) of the as-synthesized materials: (a) FDU-12, (b) Zr-in-F, (c) Zr-gra-F, (d) Zr-im-F.

Fig. 2 displays the N_2_ adsorption–desorption isotherms and pore distribution of all the as-synthesized supports. From Fig. 2A, it can be seen that all the supports show the type IV of line with a H2 hysteresis loop in the range of *P*/*P*_o_ = 0.45–0.93, which indicates that all the synthesized materials can keep mesoporous characteristics.^27^ And the sharp decrease in the range of *P*/*P*_o_ = 0.40–0.50 displays that all the materials have a relatively uniform mesopores.

It can be seen from Fig. 2B that Zr–F materials obtained by different methods show a more centralized pore distribution compared with the pure FDU-12 material. Furthermore, the pore diameters of all the materials keep the order: FDU-12 > Zr-in-F > Zr-gra-F > Zr-im-F. Textural structure parameters of the pure FDU-12 and Zr–F materials are listed in Table 1. It can be formed that the pure FDU-12 have the highest specific surface area (789 m^2^ g^−1^) and the pore volume (0.72 cm^3^ g^−1^) among all the as-synthesized materials. Furthermore Zr-in-F have the higher specific surface area and the pore volume than Zr-gra-F and Zr-im-F.^31^ It might be attributed to the fact that Zr source added in the process of post synthesis cannot be efficiently assembled into the mesoporous FDU-12 framework. After calcination, the formation of ZrO_2_ microcrystal particles resulted in the channel blockage. Thereafter, the deduction is confirmed by the characterization result of UV-Vis. Furthermore, the contents of ZrO_2_ in the supports detected by XRF characterization follows the order: Zr-im-F > Zr-in-F > Zr-gra-F.

**Table 1:** Textural structure parameters of the as-synthesized materials

Samples	BET surface area^a^ (m^2^ g^−1^)	Total pore volume^b^ (cm^3^ g^−1^)	Pore diameter^c^ (nm)	Contents of ZrO_2_^d^, *m*%
FDU-12	785	0.72	19.5	
Zr-in-F	709	0.65	18.7	8.9
Zr-gra-F	664	0.55	18.2	7.6
Zr-im-F	647	0.54	17.8	9.1

a Calculated by BET method.

b Obtained at a relatively pressure of 0.98.

c Calculated using BJH method.

d Measured by the XRF characterization.

### 3.3 SEM characterization of the supports

**Fig. 3:**
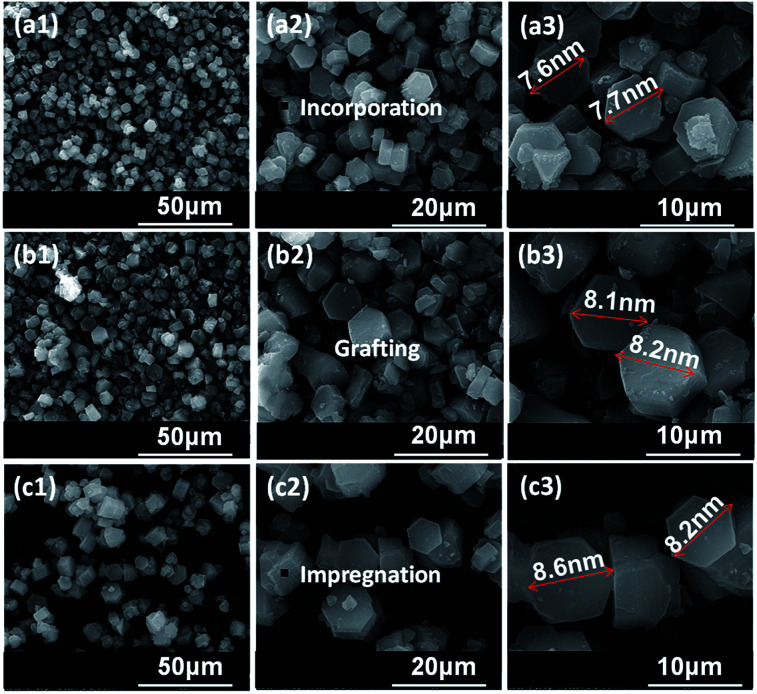
SEM images of FDU-12 series materials with different Zr modification methods: (a) Zr-in-F, (b) Zr-gra-F, (c) Zr-im-F, 1–3 represent different scales.

The morphologies of Zr–F mesoporous materials obtained by different methods are studied by SEM. It can be seen from Fig. 3 that all the synthesized Zr–F materials prepared through three different modification methods exhibit relatively regular hexagonal prims, which indicate that the morphologies of the mesoporous FDU-12 were retained after the incorporation of Zr species. Furthermore, the particle diameter of Zr-in-F is slightly less than that of Zr-im-F and Zr-gra-F.

### 3.4 TEM characterization of the supports

In order to analyze the mesoporous channels of the as-synthesized supports, all the as-synthesized materials are characterized by TEM technique, and the results are displayed in Fig. S1.† From those pictures, it can be seen that Zr-im-F and Zr-gra-F materials obtained by the post synthesized methods can keep the orderly pore structures, belonging to the typical cubic *Fm*3*m* topology of FDU-12. However, the pore structure of Zr–F material obtained by the direct synthesis method is slightly orderless comparing with those of the post synthesis. It is because the radiuses and coordination numbers of zirconium and silicon atoms are different. Thus when the silicon atoms in the framework are partially replaced by zirconium, the skeleton might be slightly twisted, leading to the decrease of the order degree.

### 3.5 FTIR characterization of the supports

**Fig. 4:**
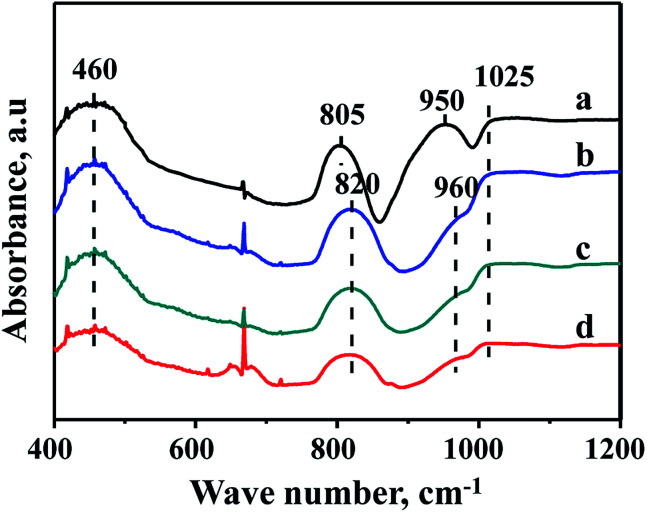
FT-IR patterns of FDU-12 series materials with different Zr modification methods: (a) FDU-12, (b) Zr-in-F, (c) Zr-gra-F, (d) Zr-im-F.

The characterization of FTIR is performed to analyze the existence state of Zr species in the supports. FTIR spectra of FDU-12, Zr-in-F, Zr-im-F and Zr-gra-F supports are displayed in Fig. 4. It can be seen from the pictures that the pure FDU-12 support shows strong bands at 460 and 805 cm^−1^, which are attributed to the bending and symmetric vibration of Si–O–Si.^32,33^ However, the band at 805 cm^−1^ shifted to 820 cm^−1^ for all the modified materials, which is attributed to the incorporation of Zr species, modulating the chemical environment of Si species. The band at 1025 cm^−1^ is the band associated with asymmetric stretching vibration of Si–O–Si bond.^34^ The pure FDU-12 material exhibits a band at 950 cm^−1^, which is assigned to Si–OH;^35^ however, the band shifts to 960 cm^−1^ for all the Zr–F materials. And the band is ascribed to the synergistic effect of the Si–OH and Si–O–Zr groups,^35^ suggesting Zr species are incorporated into the framework of all the modified materials.

### 3.6 UV-Vis characterization of the supports

**Fig. 5:**
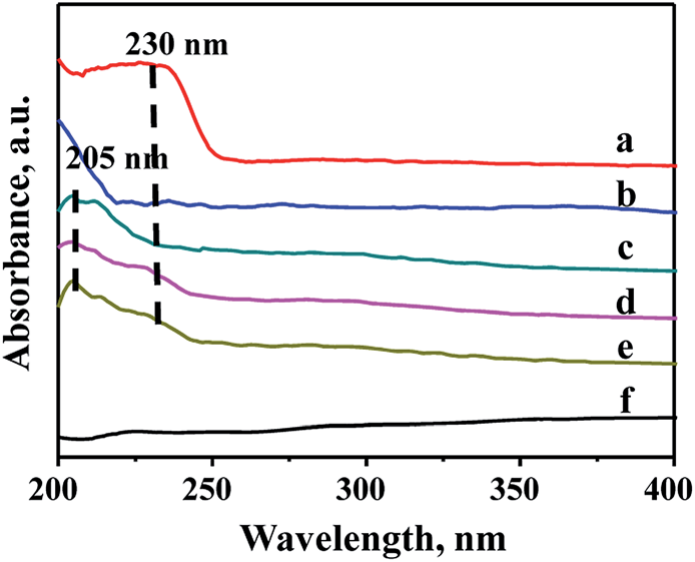
UV-Vis DRS spectra of FDU-12 series materials with different Zr modification methods: (a) ZrO_2_, (b) ZrOCl_2_·8H_2_O, (c) Zr-in-F, (d) Zr-gra-F, (e) Zr-im-F, (f) FDU-12.

**Fig. 6:**
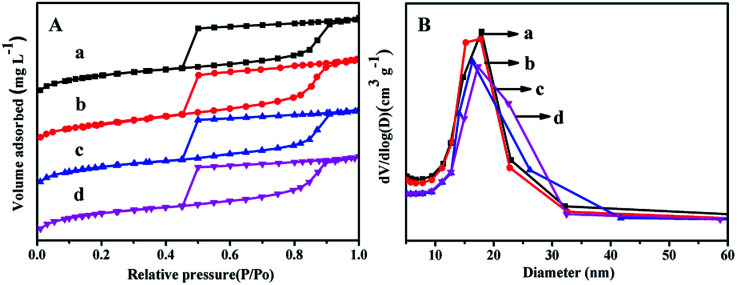
N_2_ adsorption–desorption isotherms (A) and pore distribution (B) of the catalysts: (a) NiMo/FDU-12, (b) NiMo/Zr-in-F, (c) NiMo/Zr-gra-F, (d) NiMo/Zr-im-F.

Due to the different chemical environment of the metallic species over the support, there are different coordination states for the metals, thus different absorption peaks can be observed in the UV-Vis characterization. Therefore, UV-Vis spectroscopy is widely used to characterize the modified mesoporous materials. Fig. 5 shows UV-Vis spectra of various species involving ZrO_2_, Zr source (ZrOCl_2_·8H_2_O), pure FDU-12 and Zr–F materials obtained by three different methods. It can be seen from Fig. 6 that all the modified FDU-12 materials have an obvious absorption peak at 205 nm in contrast with the pure FDU-12 material. According to the literature,^36,37^ the peak is assigned to the electron transition phenomenon from p orbit to d orbit (O→Zr) in the skeleton, which further confirms that most of Zr species have been incorporated into the framework successfully. Meanwhile, similar to ZrO_2_ spectra, Zr-im-F and Zr-gra-F also show a weak peak at 230 nm, which implies the existence of ZrO_2_.^36^ It is derived from the non-framework Zr species, which are existed as the separated phases, and absorbed on the surface of mesoporous FDU-12, then easy to transform into ZrO_2_ particles after calcination. Furthermore, the sample of Zr-in-F does not display the peak at 230 nm corresponding to ZrO_2_ bulk phases, suggested that no isolated ZrO_2_ species are formed over the support. And it can be inferred that most of the Zr species have been successfully embedded into the skeleton of mesoporous FDU-12. Combining with XRF analysis, it can also be concluded that Zr-in-F material prepared by the direct-synthesis method incorporates more Zr species into the framework than those prepared by the post-synthesis methods.

### 3.7 BET characterization of the catalyst

N_2_ adsorption–desorption isothermal and pore distribution of NiMo/FDU-12 and NiMo/Zr–F catalysts obtained by different modified methods are shown in Fig. 6. From Fig. 6A it can be found that all the prepared catalysts display the type IV isothermals with type H2 hysteresis loops, which confirm that the mesoporous texture of different materials are well maintained after impregnating active metals. It is found from Fig. 6B that all the catalysts show relatively uniform aperture distribution, and the most probable aperture of all the catalysts keep the order: NiMo/FDU-12 > NiMo/Zr-in-F > NiMo/Zr-gra-F > NiMo/Zr-im-F. The pore textures of different catalysts are summarized in Table 2. Comparing to the corresponding support materials, the specific surface area, volume and pore diameter of the catalysts slightly decrease, which are attributed to the loadings of active metals occupying some support surfaces or blocking channels.^16^

**Table 2:** Textural properties of the catalysts with different prepared methods

Samples	*S*_BET_ a (m^2^ g^−1^)	*V*_t_ b (cm^3^ g^−1^)	*d*_BJH_ c (nm)
NiMo/FDU-12	391	0.59	18.6
NiMo/Zr-in-F	373	0.43	17.8
NiMo/Zr-gra-F	358	0.39	17.2
NiMo/Zr-im-F	332	0.39	17.1

a Calculated by BET method.

b Obtained at a relatively pressure of 0.98.

c Calculated using BJH method.

### 3.8 UV-Vis of the oxide catalysts

UV-Vis spectra is performed to analyze the distribution of Mo species in different catalysts, and the results are exhibited in Fig. S2.† It is obvious from Fig. S2† that all the catalysts obtained by different methods show absorption bands at 200–400 nm, which are assigned to different coordination Mo oxide species. The band at 220–250 nm is ascribed to the isolated tetrahedral coordination species,^38,39^ and the band at 250–350 nm is ascribed to the octahedral coordination species.^39^ Furthermore, there are no absorption bands attributing to the isolated MoO_3_ species, which confirms that Mo species are well dispersed on the supports.

### 3.9 Raman of the oxide catalyst

**Fig. 7:**
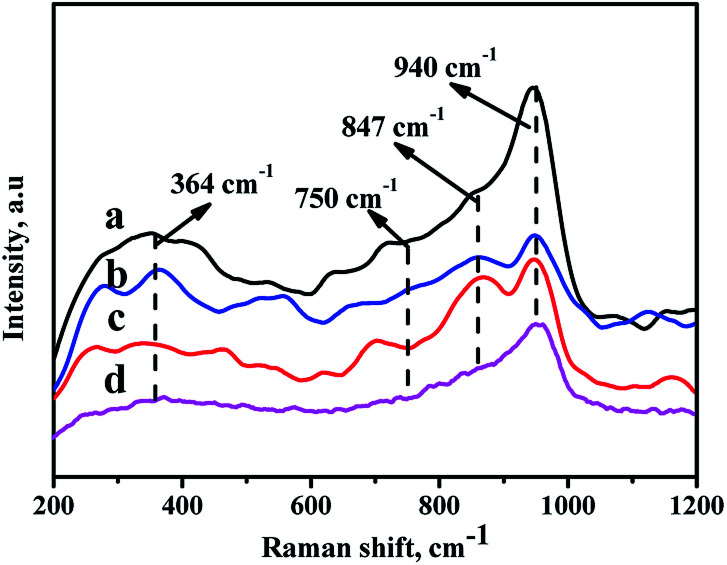
Raman spectra of the catalysts with different prepared methods: (a) NiMo/Zr-in-F, (b) NiMo/Zr-gra-F, (c) NiMo/Zr-im-F, (d) NiMo/FDU-12.

**Fig. 8:**
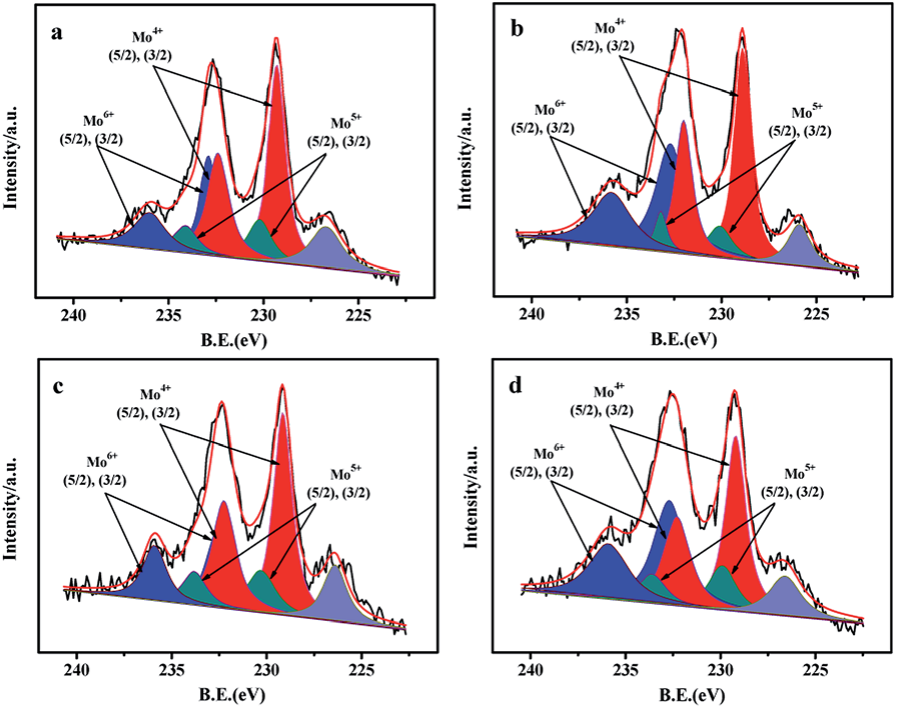
Mo 3d XPS spectra of the sulfided catalysts with different prepared methods: (a) NiMo/Zr-in-F, (b) NiMo/Zr-gra-F, (c) NiMo/Zr-im-F, (d) NiMo/FDU-12.

In order to analyze the crystalline symmetry and the nature of the oxide phases, all the catalysts are characterized by Raman spectra,^40,41^ and the results are displayed in Fig. 7. For the prepared catalysts, there are some characteristic bands at 364 cm^−1^, 750 cm^−1^, 847 cm^−1^, and 940 cm^−1^ observed in Fig. 8. All the NiMo/Zr–F catalysts exhibit a band at 750 cm^−1^ assigning to bulk Zr(MoO_4_), which is derived from the stretching vibration of Zr–O–Mo.^42^ Furthermore, the band at 364 cm^−1^ and 847 cm^−1^ are assigned to bending vibration of Mo=O in tetrahedrally coordinated molybdate (MoO_4_^2−^), and the band at 940 cm^−1^ is ascribed to the stretching vibration of Mo=O in the octahedrally coordinated polymolybdate (Mo_7_O_24_^6−^),^43,44^ which is formed due to the weak interaction between the active metals and the supports, easy to be reduced and sulfurized, resulting in high activity of hydrodesulfurization reaction. For all the modified catalysts, the intensities of bands here are in the following order: NiMo/Zr-in-F > NiMo/Zr-im-F > NiMo/Zr-gra-F. Furthermore, the catalyst NiMo/Zr-in-F does not show some bands at 289 cm^−1^, 818 cm^−1^ and 994 cm^−1^ assigned to vibration of Mo=O in MoO_3_,^45^ indicating that Mo species are highly dispersed on the surface of this catalyst.

### 3.10 XPS of the sulfided catalysts

XPS characterization is performed to obtain the information about the contents of various compounds (Zr, Mo) as well as the sulfidation degree of Mo species. The O 1s spectrums of the O element in NiMo/Zr–F catalysts obtained by different synthesis methods are presented in Fig. S3.† The vibration peaks at 532.9 and 532 eV are respectively attributed to the Si–O–Si bridge and the Si–OH bridge on the catalysts.^21^ Additionally, the less intensified peak centered at 53.09 eV is assigned to the vibration of the Si–O–Zr, indicating the presence of Zr species in the framework of FDU-12 materials.^28^

The Mo 3d XPS spectrums of the sulfided catalysts are performed to analyze the sulfidation degrees of Mo active species.^21^ From Fig. 8, the spectra of Mo 3d are decomposed into three contributions, which are Mo^4+^, Mo^5+^, and Mo^6+^ respectively. For all the catalysts, the high intensified peaks at 229.3 eV and 232.4 eV, which have a fixed intensity ratio of 3 : 2,^21,29,30^ are attributed to the Mo^4+^ 3d_3/2_ and 3d_5/2_, proving the formation of MoS_2_ species. The relatively high intensified peaks at 232.9 eV and 236.0 eV are assigned to Mo^6+^ 3d_3/2_ and 3d_5/2_, indicating that partial Mo species in oxidation states still exist on the surface of the catalysts after the sulfidation. Furthermore, the peaks of Mo^5+^ 3d_3/2_ and 3d_5/2_ with low intensities can be also observed at 230.2 eV and 233.5 eV. The bond centered at 226.7 eV is attributed to S_2S_.

The results obtained by the deconvolution method^30^ are summarized in Table 3. The sulfidation degree of Mo active species can be evaluated by using the proportion of Mo^4+^ phases in the total Mo species, while the Mo^4+^/(Mo^4+^ + Mo^5+^ + Mo^6+^) is defined as the values of Mo_sulfidation_/Mo_total_. The sulfidation degree of all the catalysts keeps the order: NiMo/Zr-in-F(56.4%) > NiMo/Zr-im-F(54.7%) > NiMo/Zr-gra-F (49.5%) > NiMo/FDU-12(45.5%), which is in agreement with the activity results of the catalysts. Finally, the XPS results demonstrate that the Mo species over the catalyst obtained by the direct synthesis are sulfided easily than those over the catalysts obtained by the post synthesis.

**Table 3:** XPS fitting results of Mo 3d spectra of the sulfide catalysts with different modified methods

Catalysts	Mo^4+^		Mo^5+^		Mo^6+^		*S*_Mo_ b, %
	ar%^a^ (229.3 eV)	ar% (232.4 eV)	ar% (230.2 eV)	ar% (233.5 eV)	ar% (232.9 eV)	ar% (236.0 eV)	
NiMo/Zr-in-F	33.5	22.9	10.1	6.8	16.0	10.7	56.4
NiMo/Zr-gra-F	29.5	19.7	7.4	4.3	23.6	16.0	49.5
NiMo/Zr-im-F	32.8	21.9	10.3	6.9	16.9	11.2	54.7
NiMo/FDU-12	25.9	19.6	10.0	6.7	22.7	15.1	45.5

a ar% means the area percentage of XPS peak.

b *S*_Mo_ = Mo_sulfidation_ = Mo^4+^/(Mo^4+^ + Mo^5+^ + Mo^6+^).

### 3.11 Py-IR of catalysts

**Fig. 9:**
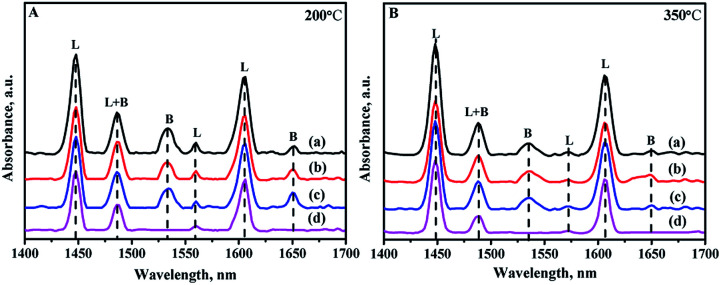
FTIR spectra of pyridine adsorbed on different catalysts after degassing at (A) 200 °C and (B) 350 °C: (a) NiMo/Zr-in-F, (b) NiMo/Zr-gra-F, (c) NiMo/Zr-im-F.

Due to the different sizes and coordination numbers of Zr and Si atoms, the introduction of Zr species into the skeleton of mesoporous silica produces electric unsaturation of Zr–O–Si, leading to the generation of acid sites, including Lewis and Brønsted acid sites.^21^ Pyridine-FTIR spectra are used to analyze the strength and types of acid sites of all the catalysts by different prepared methods. The adsorption pyridines are mainly degassed at 200 and 350 °C: the low temperature desorption at 200 °C is assigned to the total amounts of acid sites, while the high temperature desorption at 350 °C is associated with the medium and strong acid sites. Different bands can be exhibited in the wavelength of 1700–1400 cm^−1^ in the spectra as shown in Fig. 9. The bands at 1450 cm^−1^ and 1610 cm^−1^ are assigned to Lewis acid sites; and the bands at 1540 cm^−1^ and 1650 cm^−1^ are ascribed to the Brønsted acid sites, while the band at 1492 cm^−1^ is attributed to a combination of Brønsted and Lewis acid sites.^46,47^

The detailed data about the acid strength distribution and acid quantity of all the catalysts calculated from the pyridine-FTIR spectra are listed in Table S1.† It can be seen from the Table S1† that the NiMo/FDU-12 catalyst only show the existence of Lewis acid sites, and all the modified catalysts show the existence of Brønsted and Lewis acid sites, including the weak and strong acid sites. The acidities of the catalysts are associated with the incorporated quantity of Zr, and the acidities increase as the incorporation amount of Zr increases in a reasonable range of Zr species addition.^48^ The total acid amounts of all the catalysts measured at 200 and 350 °C follow the order: NiMo/Zr-in-F > NiMo/Zr-im-F > NiMo/Zr-gra-F > NiMo/FDU-12, which is in consistent with the loading of Zr species incorporated into the skeleton. This is the same to the characterization results of UV-Vis and XRF.

### 3.12 Catalytic activity

**Fig. 10:**
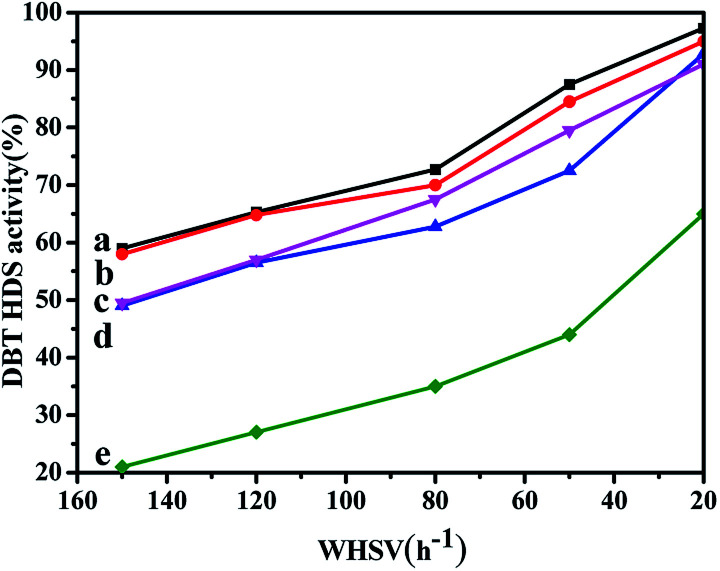
DBT HDS performance over series of catalysts with various prepared methods at different WHSV values (613 K, 4 MPa. 200 ml ml^−1^): (a) NiMo/Zr-in-F, (b) NiMo/Zr-im-F, (c) NiMo/Al_2_O_3_, (d) NiMo/Zr-gra-F, (e) NiMo/FDU-12.

The HDS activity of all the catalysts obtained by different prepared methods are evaluated using DBT (500 ppm of S, wt%) as the feedstock, and the results are shown in Fig. 10. From Fig. 10, it can be seen that the HDS performances of all the catalysts gradually increase with the WHSV values decreasing. Comparing with the NiMo/FDU-12 catalyst, the NiMo/Zr–F catalysts obtained by different synthesis routes exhibit better catalytic activities, which are attributed to higher dispersion of Mo species and more acid sites as a result of the incorporation of Zr species. The NiMo/Zr-in-F and NiMo/Zr-im-F catalysts show better catalytic activities than NiMo/Al_2_O_3_.

Among all the prepared catalysts, the NiMo/Zr-in-F catalyst show the best performance, and presents the highest DBT HDS efficiency of 97.3% at the WHSV value of 20 h^−1^. The explanation can be derived from the following reasons: firstly, the N_2_-adsorption and desorption characterizations suggested that Zr-in-F had the higher specific surface area (785 m^2^ g^−1^) comparing with the catalysts obtained by the post synthesis methods, which indicates that it can provide enough spaces for the dispersion of active sites. Moreover, the larger pore size and volume of the Zr-in-F support allowed DBT and the product molecules to diffuse rapidly in the channels. Secondly, according to the UV-Vis and Raman spectra, the Ni and Mo species were well dispersed over the NiMo/Zr-in-F catalyst, and the higher proportion of highly active multiple coordination polymolybdate species were observed over the NiMo/Zr-in-F catalyst in Fig. 7, which facilitated to increase the HDS performance. Furthermore, the catalyst NiMo/Zr-in-F had the most sulfided molybdenum species as shown in Table 3 compared with the other catalysts. Finally, the NiMo/Zr-in-F catalyst possessed more acid sites from the data in Table S1,† which would be advantage to break the C–S bond and transfer alkyl groups. The performances of all the prepared catalysts kept the following order: NiMo/Zr-in-F > NiMo/Zr-im-F > NiMo/Al_2_O_3_ > NiMo/Zr-gra-F > NiMo/FDU-12. For the NiMo/Zr-gra-F catalyst, although it had larger specific surface area and pore size, the less acid sites and the sulfided degree restricted the HDS reaction. Thus, NiMo/Zr-gra-F exhibited a lower catalytic activity than that of NiMo/Zr-im-F. In conclusion, the NiMo/Zr-in-F catalyst with higher specific surface, pore volume, pore size, and more acid sites, displayed the highest DBT HDS performance.

## 4. Conclusions

A series of Zr-FDU-12 materials were synthesized by three different methods, including the direct synthesis and post synthesis (grafting and impregnating) routes, and the corresponding catalysts were prepared by impregnation with Mo and Ni active metals. Then the materials and catalysts were characterized well *via* different techniques, *i.e.* the SAXS, Py-IR and XPS. According to the results of SAXS and TEM analysis, all the modified materials maintained a relatively ordered mesoporous structures after the incorporation of Zr. The N_2_ adsorption and desorption results showed that the Zr-in-F material obtained by the direct synthesis method possessed the largest specific surface area (709 m^2^ g^−1^), volume (0.65 cm^3^ g^−1^) and pore size (18.7 nm) in contrast with the Zr-im-F and Zr-gra-F materials. Meanwhile combining with the FT-IR and UV-Vis characterizations, Zr species were well incorporated into the framework of Zr-in-F material comparing with Zr-gra-F and Zr-im-F materials. Furthermore, based on the Raman and UV-Vis characterization results, it could be found that all the modified catalysts had better dispersion of Mo active phases. The catalytic performances of all the catalysts were evaluated in hydrodesulfurization using DBT as the feedstock, which demonstrated that the catalyst NiMo/Zr-in-F had the highest DBT HDS activity among all the prepared catalysts. It could be deduced that the good catalytic performance of NiMo/Zr-in-F was ascribed to the synergistic influence of its larger specific area (373 m^2^ g^−1^), pore volume (0.43 cm^3^ g^−1^), pore size (17.8 nm), and more acid sites (104.8 mmol g^−1^), which conform it a more potential catalyst for HDS process.

## Conflicts of interest

No conflict of interest exists in the submission of this manuscript, and the manuscript is approved by all authors for publication.

## Supplementary Material

RA-008-C8RA05032E-s001
