# Supplementary material for: Synthesis of zirconium modified FDU-12 by different methods and its application in dibenzothiophene hydrodesulfurization
Source: RSC Adv. 2018 Aug 2;8(48):27565–73. doi: 10.1039/c8ra05032e (PMC9083899; doi:10.1039/c8ra05032e)
Supplement: RA-008-C8RA05032E-s001 [file RA-008-C8RA05032E-s001.pdf]

Electronic Supplementary Material (ESI) for RSC Advances.
This journal is © The Royal Society of Chemistry 2018

Supporting Information

Synthesis of Zirconium modified FDU-12 by different methods and their application in DBT HDS process

Qian Meng, Peng Du, Bo Wang, Aijun Duan*, Chunming Xu*, Zhen Zhao, Cong Liu, Di Hu, Yuyang Li, Chengkun Xiao

State Key Laboratory of Heavy Oil Processing, China University of Petroleum, Beijing, 102249, P. R. China.

*Corresponding authors, E-mail: duanaijun@cup.edu.cn; xcm@cup.edu.cn.

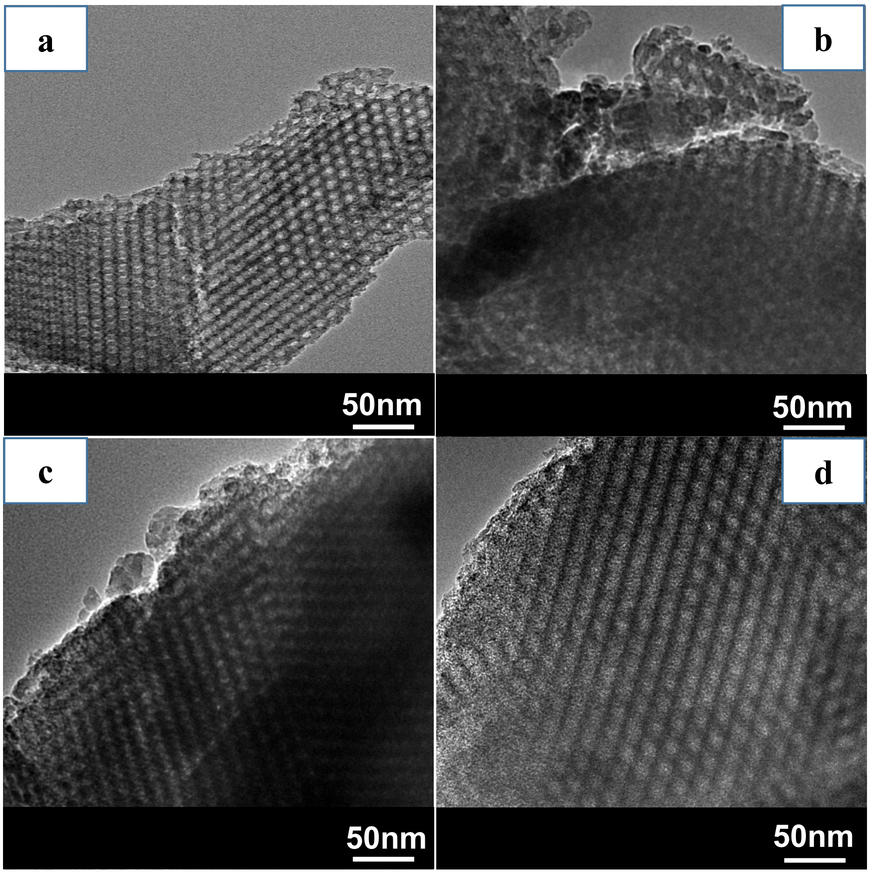

Figure S1. TEM images of FDU-12 series materials with different Zr modification methods: (a) FDU-12, (b) Zr-in-F, (c) Zr-gra-F, (d) Zr-im-F.

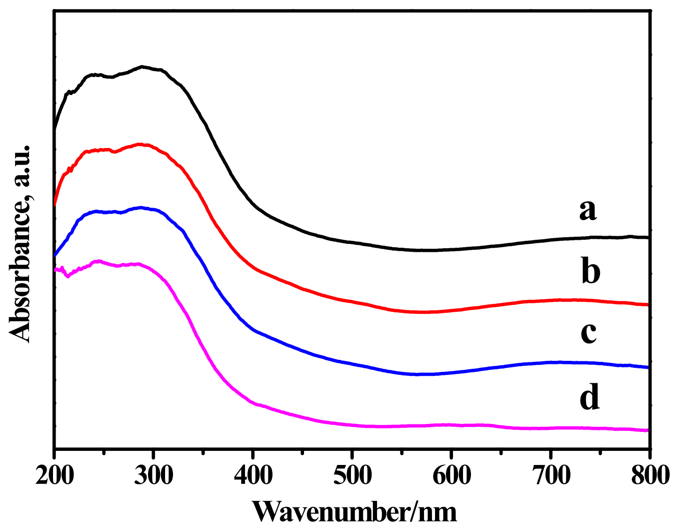

Figure S2. UV-Vis DRS spectra of the catalysts with different prepared methods: (a) NiMo/FDU-12, (b) NiMo/Zr-in-F, (c) NiMo/Zr-gra-F, (d) NiMo/Zr-im-F.

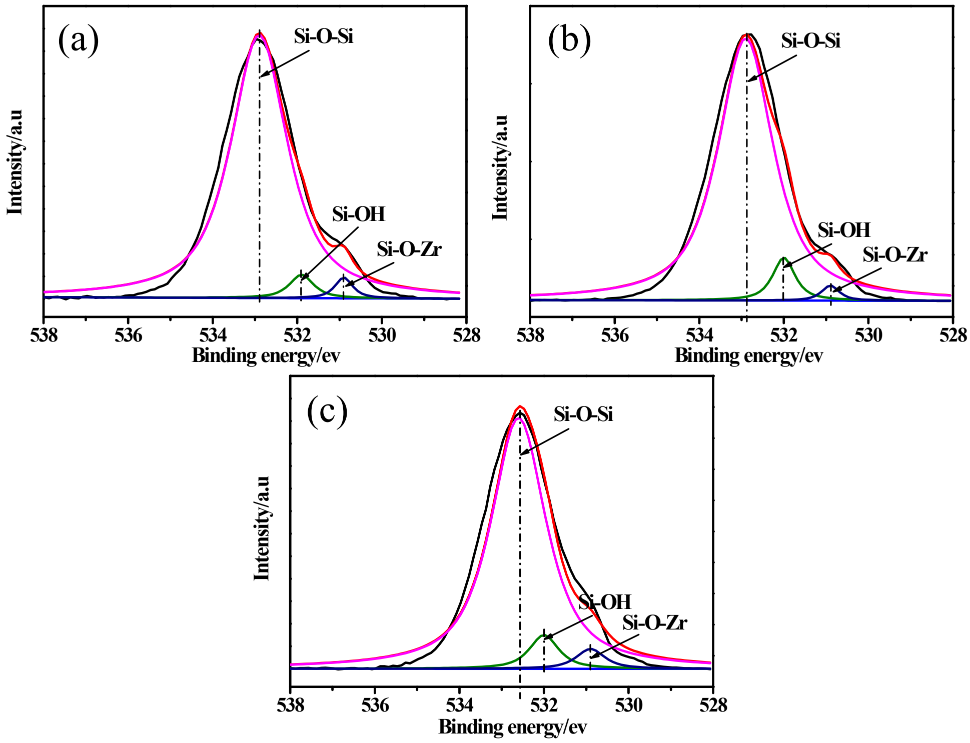

Figure S3. XPS spectra of the sulfide catalysts with different prepared methods: (a) NiMo/Zr-in-F, (b)NiMo/Zr-gra-F, (c) NiMo/Zr-im-F.

Table S1 Surface acid amounts and acid types over different catalysts.

Catalysts	Acid amount (200 °C)/mmol·g^{-1}				Acid amount (350 °C)/mmol·g^{-1}			
	L	B	L+B	B/L	L	B	L+B	B/L
NiMo/Zr-in-FDU-12	87.4	17.4	104.8	0.12	28.6	10.6	41.1	0.37
NiMo/Zr-gra-FDU-12	63.8	16.6	80.4	0.21	18.0	8.7	26.7	0.48
NiMo/Zr-im-FDU-12	78.0	18.6	96.6	0.24	25.4	11.1	36.5	0.44
NiMo/FDU-12	56.5	0	56.5	0	15.9	0	15.9	0

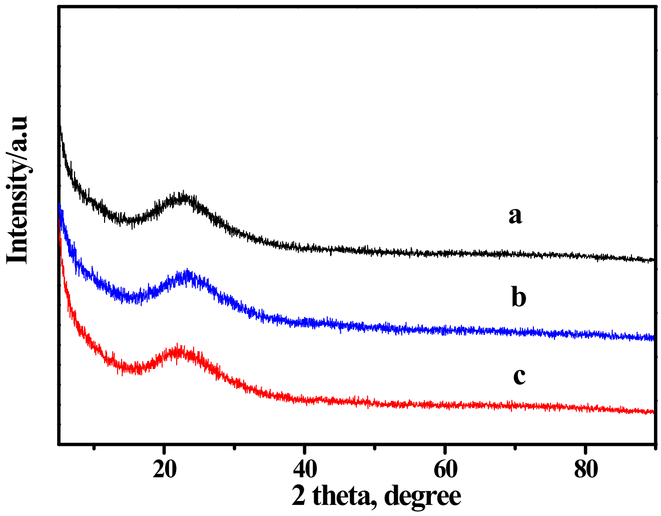

Figure S4. Wide angle XRD patterns of various materials: (a) Zr-in-F, (b) Zr-im-F, (c) Zr-gra-F.

Wide angle XRD characterizations of the series materials are shown in Fig S4. It can be observed from Fig S4 that all the materials do not display the characteristic peaks of tetragonal and monoclinic ZrO_2, indicating that Zr species are well dispersed on the supports or the dimensions of ZrO_2 clusters are too small to be de detected.

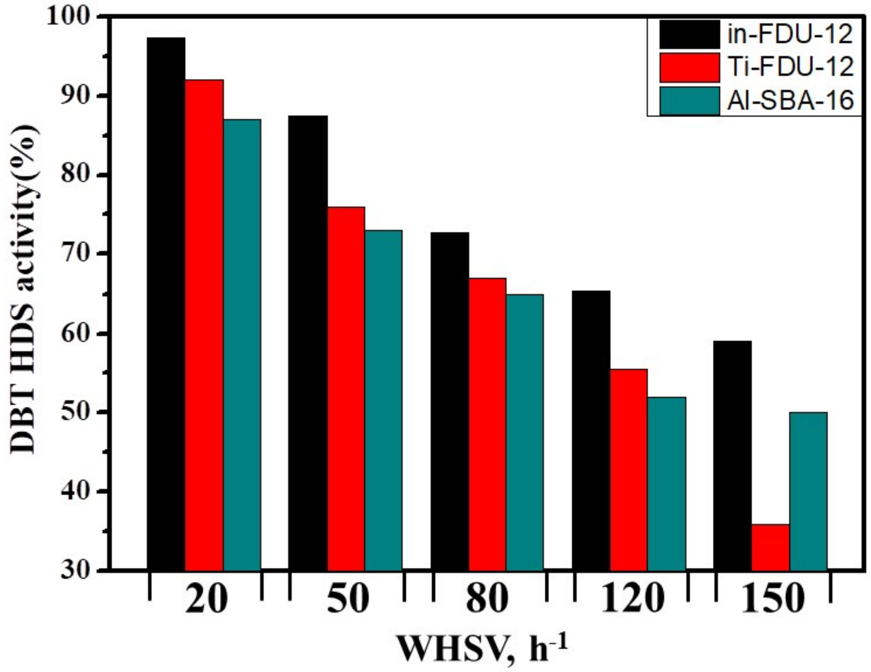

Figure S5. DBT HDS performance over various catalysts at different WHSV values (340 °C, 4 MPa, 200 ml/ml)

According to the literatures[1-2], the NiMo/Ti-FDU-12 and NiMo/Al-SBA-16 catalysts were obtained.

[1] ZK Cao, AJ Duan, Z Zhao, JM Li, YC Wei, GY Jiang, J Liu. A simple two-step method to synthesize the well-ordered mesoporous composite Ti-FDU-12 and its application in the hydrodesulfurization of DBT and 4,6-DMDBT[J]. Journal of Materials Chemistry A, 2014, 2(46):19738-19749.

[2] ST Song, XF Zhou, AJ Duan, Z Zhao, KB Chi, MH Zhang, GY Jiang, J Liu, JM Li, XL Wang. Synthesis of mesoporous silica material with ultra-large pore sizes and the HDS performance of dibenzothiophene[J]. Microporous & Mesoporous Materials, 2016, 226:510-521.
